# The Study of Copper Powder Sintering for Porous Wick Structures with High Capillary Force

**DOI:** 10.3390/ma16124231

**Published:** 2023-06-07

**Authors:** Im-Nam Jang, Yong-Sik Ahn

**Affiliations:** Department of Materials Science and Engineering, Pukyong National University, Busan 48547, Republic of Korea; imnam12@naver.com

**Keywords:** sintering, wick, porosity, permeability, capillary force

## Abstract

The porosity, permeability, and capillary force of porous sintered copper were examined in relation to the effects of copper powder size, pore-forming agent, and sintering conditions. Cu powder with particle sizes of 100 and 200 μm was mixed with pore-forming agents ranging from 15 to 45 weight percent, and the mixture was sintered in a vacuum tube furnace. Copper powder necks were formed at sintering temperatures higher than 900 °C. The porosity, as determined by the Archimedes measurement method, and the permeability performance of the sintered body displayed higher values when the Cu powder size was uniform or small. To investigate the capillary force of the sintered foam, a test was conducted using a raised meniscus test device. As more forming agent was added, the capillary force increased. It was also higher when the Cu powder size was larger and the size of the powders was not uniform. The result was discussed in relation to porosity and pore size distribution.

## 1. Introduction

Recently, the functional requirements of electronic equipment have become more complex as a result of the rapid development of the IT and electronics industries and the high-power consumption of that equipment. [1]. Heat pipes were initially created to control the temperature of satellites, but they are now used in a variety of fields and applications for the electronics industry, including phase-change heat transfer with super thermal conductivity, a wide range of automatic temperature characteristics, guarantee of long lifespan and price competitiveness, etc. 

Porous copper wicks are especially commonly used in heat pipes; they have higher thermal conductivity and hence effectively dissipate heat better. Generally, porous copper metal has a high strength to weight ratio with good foam mechanical energy absorption during compression, which is contributed by the ductility. It also offers several interesting features such as low density, high stiffness, high gas permeability, and high thermal conductivity. The excellent thermal and electrical conductivities of the copper foam are ideally suited for a wide range of applications such as heat exchanger and catalyst support. 

The working fluid in heat pipes is pumped from the condensing portion to the evaporation section and back again by the copper wicks, which are mounted on the inner wall of the copper pipes [2]. Therefore, it is important to consider the pore characteristics of sintered copper wick structures. In this study, PMMA (polymethyl methacrylate) was first used as an organic pore-forming agent to introduce pores into the copper sintered body. However, copper foam pores collapsed before a neck between the copper powders could form because PMMA’s melting point is much lower than copper powder’s sintering temperature (800 °C). It was challenging to create open pores that are uniform and homogeneous.

Lost carbonates sintering (LCS) is one of the solid route technologies to create open-cell porous metals with controlled pore structures. LCS is a simple and inexpensive process and enables control over pore size and porosity. Porous metal parts can be obtained by removing the carbonate particles from the sintered compact either by decomposition or dissolution. The sintering-dissolution process (SDP) is employed when there are two phase precursors and one phase is water soluble [3].

The LCS technique and SDP process are used in this study to create open-pore cell Cu foam. According to several researchers [4,5,6], potassium carbonates (K_2_CO_3_) are suitable carbonates to be used as pore-forming agents. This is because K_2_CO_3_ is soluble in water and has a melting point that is higher than the temperature at which copper begins to sinter. The wick structure’s capillary force, which is mainly correlated to the porosity, permeability, and thickness of the sintered body after sintering and is strongly dependent on the size and form of the metal powder used for sintering, is especially significant [7,8,9]. This study is based on investigating the relationship between the porous copper structure’s capillary property and its porosity and permeability in the presence of various copper powder sizes and pore-forming additions.

## 2. Materials and Methods

Three different types of copper powder were used to make copper foam samples. Two types of powders had the same size of 100 μm, in which the size uniformity was very different: one was very uniform in size and the other was not. The third type of Cu powder has a different particle size of 200 μm. The pore-forming agent was K_2_CO_3_ which had a globular shape and an average size of 35 μm. Copper powder was mixed with a pore-forming agent at a weight percentage of between 15 and 40 percent, compacted, and then sintered. The weight percent of the pore former is calculated to the volume percentage and the result is listed in Table 1.

**Figure 1:**
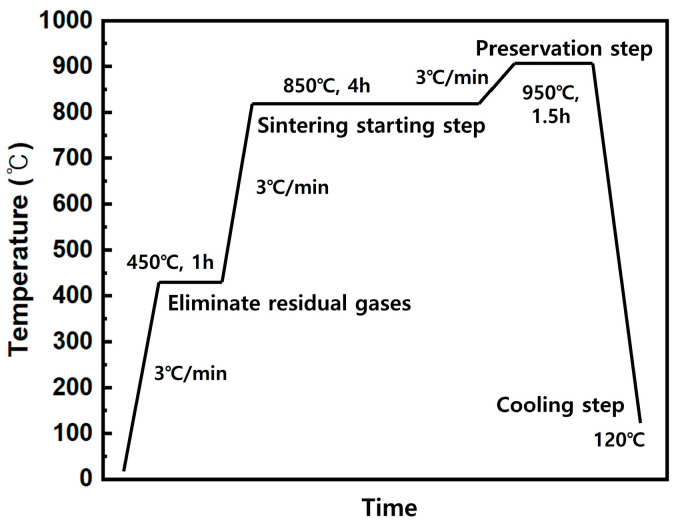
Schematic illustration of the heat schedule for sintering.

The powder mixture was sintered, as shown in Figure 1. The hydrogen was charged at a flow rate of 5 cc/min after being heated to 450 °C at a rate of 3 °C/min in a vacuum atmosphere (760 Torr). The temperature was increased to 850 °C in the hydrogen-reducing atmosphere, maintained there for four hours, and then increased to 950 °C, which was maintained there for an hour and a half [10]. The cylindrical, 10 mm × 36 mm-sized porous foams were sintered. The cross section of sintered samples was observed using an OM (optical microscope) and the formation of necks between copper powders was investigated by SEM (Scanning Electron Microscope).

**Figure 2:**
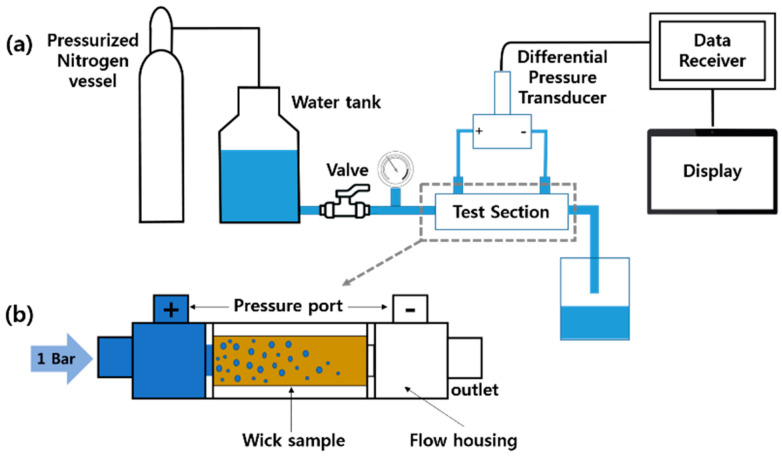
Schematic view of the permeability test apparatus: (**a**) test system and (**b**) cross-section of the test section.

Figure 2 shows a schematic illustration of the permeability test apparatus. It measured the difference in water pressure between the sample’s entrance and outflow (the beginning pressure was 1 bar). The low differential pressure indicates the high transmittance of the sample. For the test, distilled water was utilized and the water tank was pressurized with nitrogen gas. After thoroughly sealing the sample inside the cylindrical SUS 304 flow housing, the test was carried out. To ensure that water did not leak between the sample and the housing and that all water entering the inlet flowed through the outlet, Teflon tape was circumferentially wrapped around the sample and firmly affixed to the interior of the housing. The differential pressure sensor (Gilgtron, 0–1 bar), which is linked to the housing’s inlet and outlet, communicates data as water flows through the housing. The data receiver then adjusts the value (collects 200 data points per second and calculates the average value) and displays the corrected value. The test temperature was about 26 ± 0.1 °C.

The laminar flow was valid and the inertial effect was disregarded because the liquid flow was in a steady state. According to Darcy’s Law, permeability (K) can be calculated as follows.

(1) $$K=\frac{v\mu_{l}L}{∆P}$$

where $\mu_{l}$ is the viscosity of the fluid, *L* is the length of the sintered body, ΔP indicates the pressure drop, and v is the fluid’s velocity to be determined by the formula.

(2) $$v=\frac{m}{A_{w}\rho_{l}}$$

where *m* is the mass flow rate, $A_{w}$ is the test body’s cross-sectional area, and $\rho_{l}$ is the density of the liquid [11].

**Figure 3:**
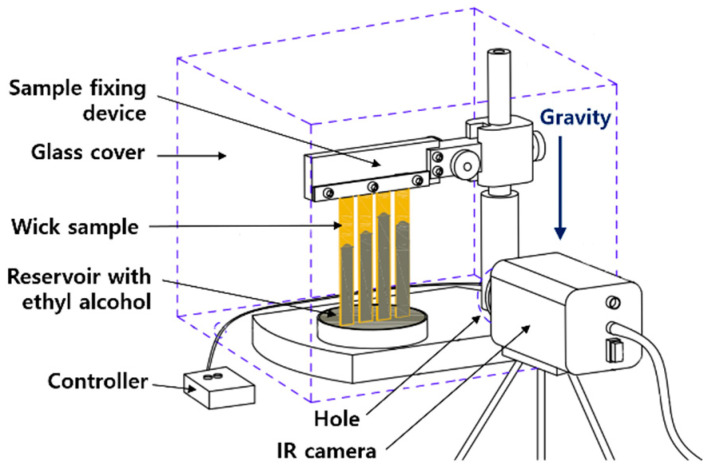
Schematic view of the capillary rate-of-rise test apparatus.

By monitoring the meniscus height and the rising rate in a piece of capillary experiment equipment, the capillary force of a sintered foam sample was determined. To evaluate the capillary force of porous surfaces, a raised meniscus test device was constructed as shown in Figure 3. Sintered cylindrical form samples with a length of 36 mm were mounted vertically on a jig and an IR camera was used to measure the height at which ethanol was raised as the bottom of the samples was allowed to touch the liquid. A glass cover was employed to provide a consistent airflow in the situation and the ambient temperature was 26 °C. The height of the meniscus was determined for the image of ethyl alcohol absorption [11,12].

## 3. Results

### 3.1. Powder Sintering and Porosity

**Figure 4:**
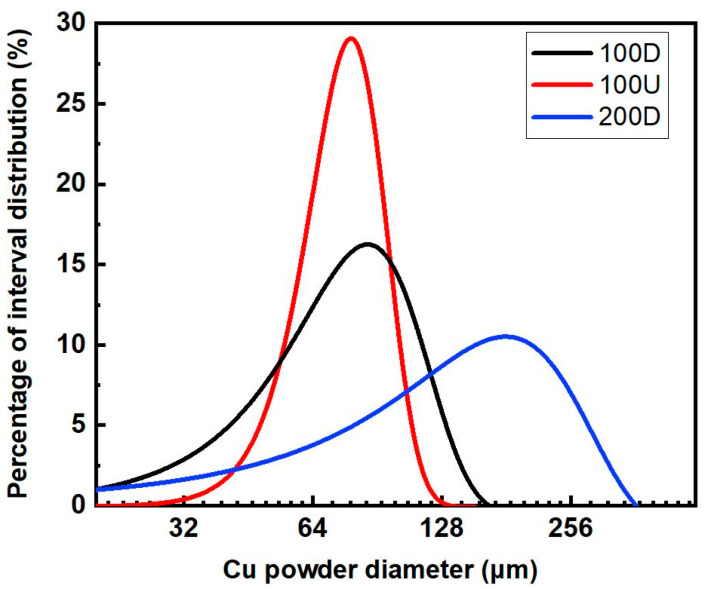
Size distribution of spherical copper powders.

The size distribution of three different types of copper powders is shown in Figure 4, where 100D and 100U powders have a similar average size of about 100 μm despite having very different size uniformities. The 100U powder has a relatively uniform size compared to 100D powder, which has a widely distributed powder size. The average particle size of 200D powder is 200 μm, although the size distribution is not homogeneous.

**Figure 5:**
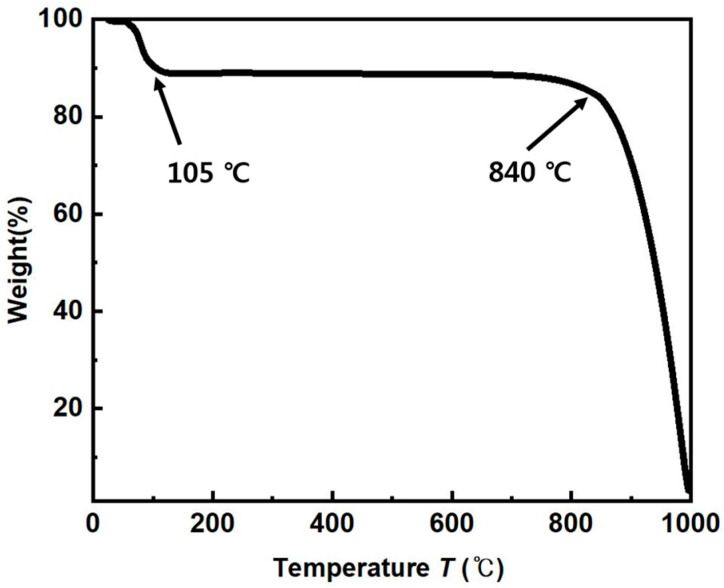
Weight changes of a pore former of K_2_CO_3_ during heating using a TGA analyzer.

A TGA (thermos gravimetric analysis) test was carried out to ascertain the mixture’s sintering temperature and the result is displayed in Figure 5. According to the TGA data, the pore-forming agent of K_2_CO_3_ began to thermally decompose at 840 °C and burned out at about 1000 °C. As a result of the fact that the temperature at which the pore former decomposes, 840 °C, is higher than the temperature at which Cu powder begins to sinter, this indicates that the pore former is suitable for producing a porous body. At a temperature of approximately 800 °C is when the Cu powders start to sinter and at around 850 °C to 950 °C is when the process is finished. As a result, during sintering, the porous structure can continue to exist without collapsing. Pores remain in the space where the pore former once was after sintering [13].

**Figure 6:**
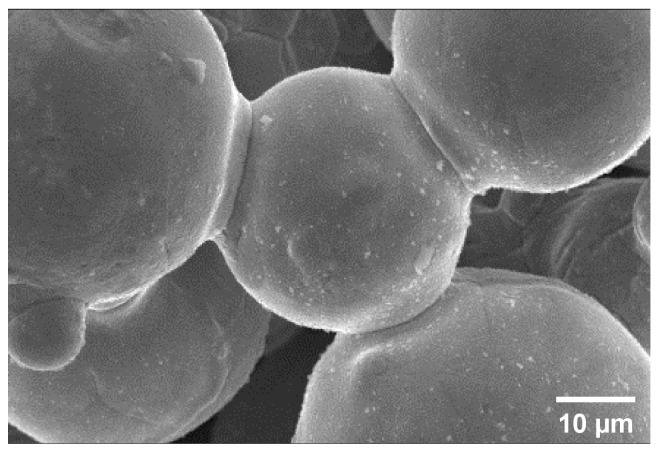
SEM photograph of the sintered Cu 200D powder.

The SEM micrograph of the sintered 200D Cu powders in Figure 6 shows the initial stage of solid phase sintering. Due to the reduction atmosphere of H_2_ during sintering, the surface and neck of powders are well formed and have a large number of open pores without oxidizing.

The porosity (*ε*) was determined by applying the Archimedes principle. For this, the weight (*W_dry_*) of completely dried samples was obtained using weight measurement after one hour of boiling in distilled water and 24 h of cooling, and the saturated mass (*W_solv_*) was acquired. *W_wet_* denotes the gauged mass in liquid. Thus, the porosity (*ε*) was determined by using the Equation (3) [2].

(3) $$\varepsilon =\frac{W_{wet}-W_{dry}}{W_{wet}-W_{solv}}\times 100\left(\%\right)$$

**Figure 7:**
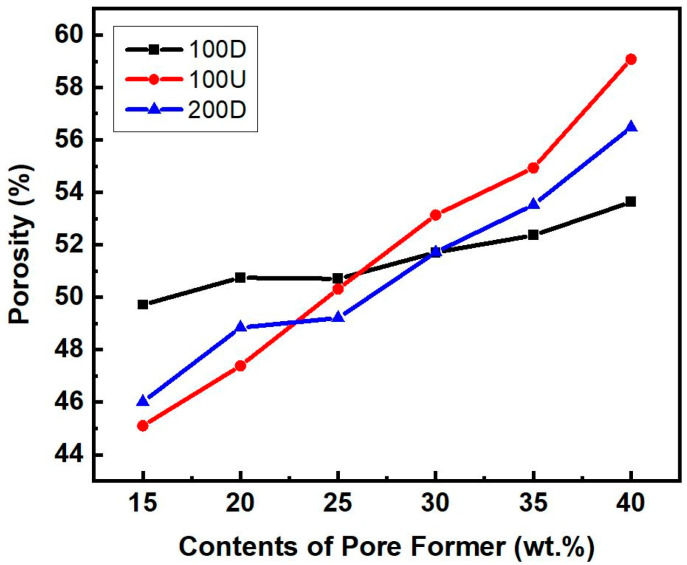
The measured porosity using Archimedes’ principle in relation to the amount of pore-former and the type of copper powder.

Figure 7 shows that the porosity (*ε*) generally increases as the contents of pore former increase. Among the three powders with different distributions, 100D had the highest porosity when the pore former content ranged from 15 to 20 wt.%, whereas it showed the lowest when the content ranged from 35 to 40 wt.%. In comparison to the powders of 100U and 200D, 100D is less affected by the porosity by the additional amount of the pore former. 

Combining fine and coarse particles effectively affects the filling rate, and the natural density of the powder is also influenced by these factors. This is because the first filling rate, which occurred before the intermediate sintering stage, had a significant effect on the porosity [14].

In comparison to the 100U sample, the 100D and 200D samples had non-uniform particle sizes and significantly more tiny particles, hence their packing density should have been higher. The 100U sample had a higher porosity due to the low packing density. When pore former was added at a 40 wt.%, the 100U sample showed the highest porosity of the three, whereas the 100D sample had the lowest porosity. This should be attributed to the fact that powder particle gaps during sintering tended to be filled or covered by other smaller particles. As a result, the foam has a lot of closed pores. Surface energy per unit weight is typically higher for smaller powder particles than for larger powder particles [15]. As a result, it was expected that 100D would have a higher density than the uniformly sized 100U sample and that the porosity of 100U would be more affected by the contents of the pore former.

### 3.2. Permeability

**Figure 8:**
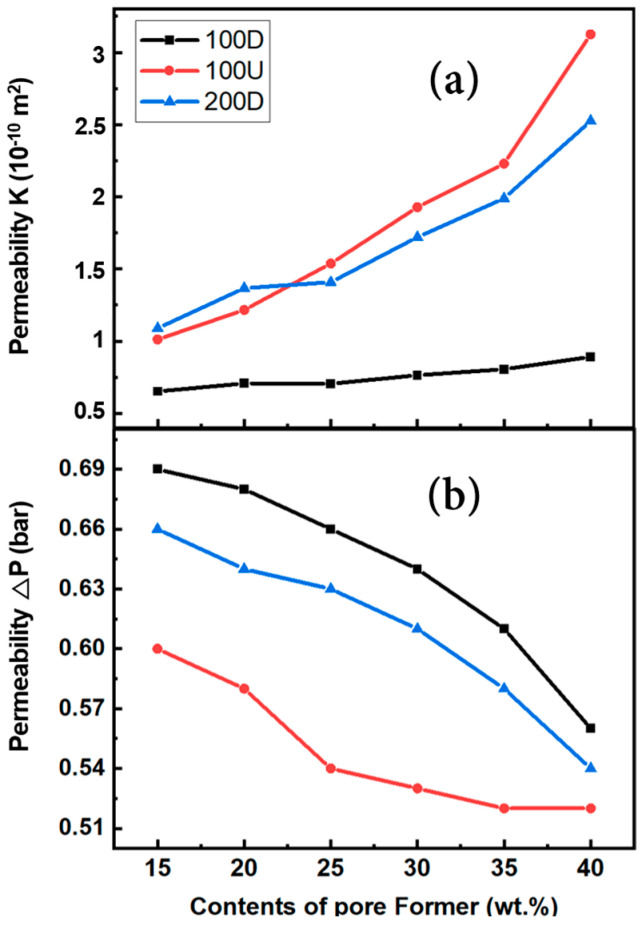
The estimated permeability (**a**) with porosity and the observed permeability (**b**) using a device of three different types of powders in relation to the contents of the pore former.

The permeability (K) using the measured porosity can be simply determined by Equation (4) [16]:

(4) $$K=\frac{d^{2}\varepsilon^{2}}{150{\left(1-\varepsilon \right)}^{2}}\left(m^{2}\right)$$

where *ε* is the porosity and *d* is the average particle size. Figure 8a shows the permeability estimated using Equation (4) with the porosity. With an increase in pore former content, which also led to an increase in porosity, permeability increased. Between 25 to 40 wt.% of pore former content, the 100U sample generally exhibited the highest permeability, whereas the 100D sample exhibited the lowest values, similarly to the porosity rise with the pore former, as shown in Figure 6. The permeability measured using a permeability device is shown in Figure 8b as the difference in water pressure between the housing’s inlet and outlet. ΔP denotes the variance in water pressure. A lower ΔP value suggests higher permeability, whereas a higher ΔP value indicates a larger differential pressure and, as a result, lesser permeability. 

The results are in good agreement with the porosity-based permeability value shown in Figure 8a. Regardless of the amount of pore former, 100U showed the highest permeability while 100D showed the lowest value. This should be connected to the higher porosity due to the uniform particle size in the 100U samples. When compared to the 100D sample, the 200D sample with larger particles showed higher porosity and permeability, but lower porosity and permeability than the 100U sample. Within the scope of this study, the uniformity of the particle size had a more significant influence on the porosity and permeability than the particle size.

### 3.3. Capillary Force

We can calculate the capillary capacity (Δ*P_total_*) by using the wetted height and the rising velocity of the wetted height of the meniscus. Since the total pressure loss is equal to the capillary force [15], the friction pressure loss and the hydrostatic pressure loss, which are two components of the total pressure loss, can generally be combined to get the following Equation (5) [17]:

(5) $$∆P_{total}=\frac{\mu \varepsilon }{K}h\frac{dh}{dt}+\rho gh$$

where μ is the viscosity of the working liquid, ε is the porosity of porous structure, *K* is the permeability of porous structure, *h* is the wetted height, *dh*/*dt* is the rising velocity of the wetted height, ρ is the liquid density, and *g* is the gravitational acceleration. Due to the marginal inertial effects, the second term of *ρgh* can be ignored, leaving the liquid rise as the only notable effect on the capillary capacity. The following Equation (6) can therefore be used to estimate the capillary force [17]:

(6) $$∆P_{cap}=\frac{\mu \varepsilon }{K}h\frac{dh}{dt}$$

**Figure 9:**
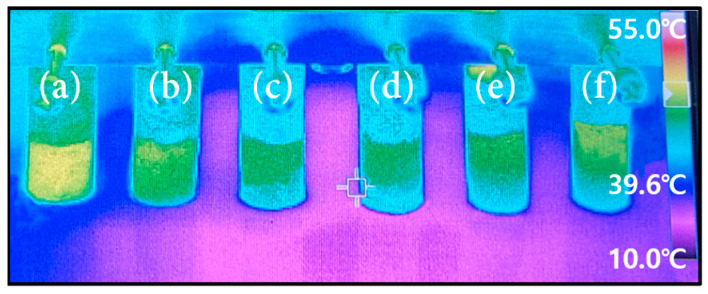
IR images of the alcohols’ menisci rising behaviors in the Cu porous 100U samples with different pore former contents. (**a**) 15 wt.%, (**b**) 20 wt.%, (**c**) 25 wt.%, (**d**) 30 wt.%, (**e**) 35 wt.%, (**f**) 40 wt.%.

**Figure 10:**
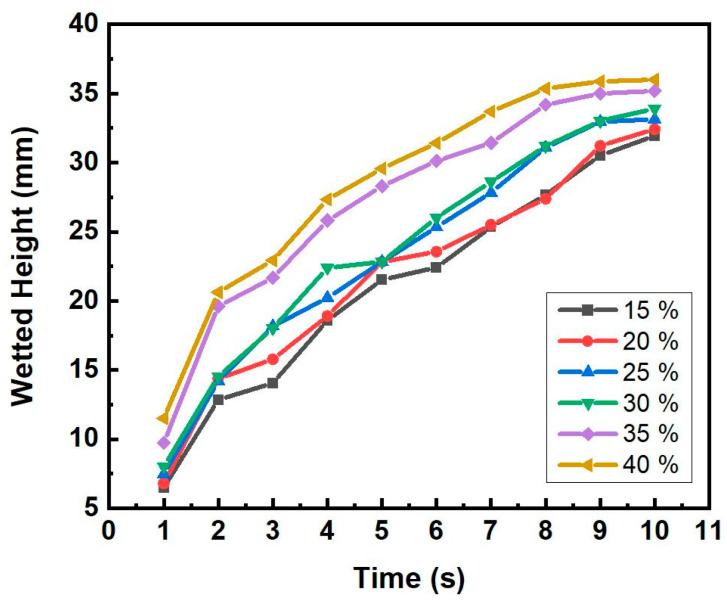
The capillary height rises with time in the sintered 200D sample with various pore former contents.

Figure 9 shows the image of the alcohols’ menisci rising behavior in the 100U samples, which had pore former contents ranging from 15 to 40 wt.%. Using an Infrared camera, in terms of temperature contrast, the menisci’s rising heights were thought to be the shortest lengths of the green or yellow sections and the heights were recorded every second. The image illustrates how the height of alcohol changes over time. We may compare the capillary force by measuring the alcohol height (h) and rising speed (*dh*/*dt*) as the capillary force is proportional to both according to Equation (6). The measured capillary rise height (h) of 200D samples with various forming agent contents is shown in Figure 10 as a function of time (s).

**Figure 11:**
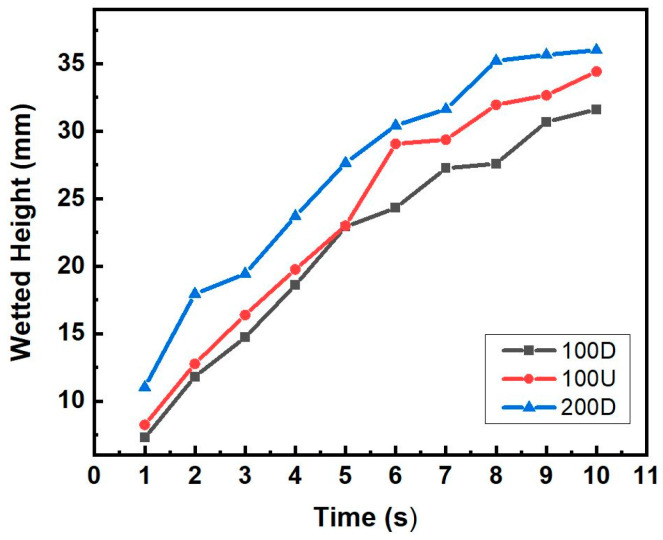
The capillary height rises with time in 3 different samples with pore former of 40 wt.%.

The capillary rise height generally rose with increased pore former contents and wetting time. The porosity of the sample increased with the addition of the pore former as shown in Figure 6, which in turn could increase capillary force. Figure 11 shows the capillary height variation of the samples sintered with various Cu powders with the same 40 wt.% pore former content. 

The 200D sample, followed in that order by the 100U and 100D samples, showed the highest rising speed (dh/dt) of wetted height from the beginning. Although the 100U and 100D samples showed similar rising speeds in the beginning, after 5 s, 100U has a noticeably higher speed than 100D. This result does not correspond with the result of the porosity measurement. The samples with the same 40 wt.% pore former content showed the porosity proportion order of 100U, 200D, and 100D, as shown in Figure 7. Figure 8 showed that the measured permeability value was in the same order as the porosity proportion of the 100U, 200D, and 100D samples; however, the capillary force of the 200D, 100U, and 100D samples was in a different order. This suggests that it is not possible to only predict the capillary force based on the porosity and permeability of porous bodies. The specimens could exhibit varying capillary forces despite having the same porosity because, as Meyers et al. [18]. reported, capillary force can be affected by other factors such as the size, shape, and connectedness of the pores. The capillary force (Δ*P_cap_*) can also be calculated using the Laplace-Young equation, as illustrated in Equation (7) [19]:

(7) $$∆P_{cap}=\frac{2\sigma }{R_{eff}}$$

where *σ* is the liquid’s surface tension and *R_eff_* is the effective pore radius. Hence, the sintered specimen with a smaller effective pore radius is thought to have a higher capillary force under the same surface tension conditions [19]. We therefore attempted to measure the effective pore radius of each specimen.

**Figure 12:**
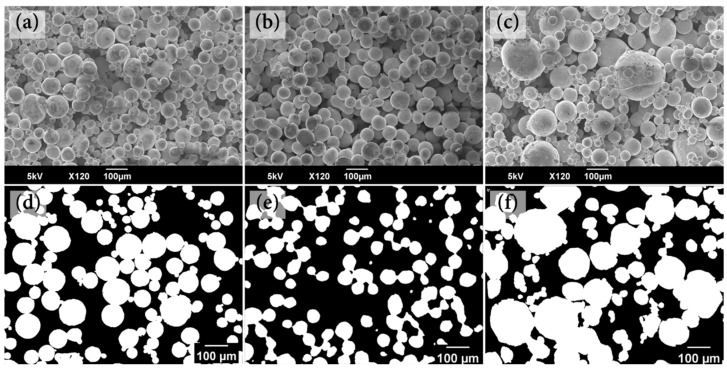
SEM photograph and optical microstructures of porous bodies sintered from several types of Cu powder with the same 40 wt.% pore-former content. (**a**,**d**) 100D, (**b**,**e**) 100U, (**c**,**f**) 200D. (white: copper powder and black: pore).

Figure 12 shows the SEM (a, b, c) and optical micrographic images (d, e, f) of samples sintered with 100D, 100U, and 200D particles, all of which had a 40 wt.% pore former content. The optical micrographic images (d, e, f) are transformed images by the image analysis program to set a proper threshold to the gray region to extract objects (pores) from their back-bound depending on the strength of the object and backdrop contrast [20]. The white area denotes sintered particles, whereas the gray area is a porosity. 

The microstructure of sample 100D, which was sintered from mixed-sized particles having an average particle size of 100 μm, is shown in Figure 11a,d. Compared to the 100D sample, the 100U sample (Figure 11b,e) contains more large pores but fewer small pores because the particle sizes are more uniform. As a result of the sintering of mixed-sized particles, the 100D sample exhibits large-sized pores along with some small-sized closed pores. The forming agent should have been positioned in the large pores prior to sintering, while the small, closed pores should have been sealed up during the sintering of the particles. The 200D sample (Figure 12c,f) was sintered from a mixture of particle sizes with an average particle size of 200 μm; it exhibits large and small pores. Small, closed pores cannot affect capillary action or permeability. Hence, we determined the average effective pore radius of open pores which make a real impact on the capillary action of each sample. Table 2 shows the average effective pore size (*R_eff_*), porosity (*ε*), and measured permeability (Δ*P*) of 100U, 100D, and 200D samples sintered with the same 40 wt.% pore former content. The 100U sample has the largest *R_eff_* value of 241.31 μm, followed by 100D and 200D in that order. According to Equation (5), the effective pore radius (*R_eff_*) and the capillary force (Δ*P_cap_*) are inversely related, indicating that the capillary force is stronger with a smaller R_eff_.; consequently, the order of the capillary force should be 200D, 100D, and 100U samples in that order. Table 2 showed that the 200D sample had the biggest capillary force (Δ*P_cap_*) due to the smallest *R_eff_*, as expected.

Despite the fact that the 100U sample had a larger *R_eff_*, the 100U sample showed a larger Δ*P_cap_* than the 100D sample in the comparison between the two samples. This should be due to the 100U sample’s larger porosity. This suggests that the capillary force (Δ*P_cap_*) is influenced in a complex way by the porosity (*ε*) and the refractive factor (*R_eff_*). The capillary force (Δ*P_cap_*) increased with Equation (6) with an increase in porosity (*ε*) and it also increased with a decrease in pore size as in Equation (7). More investigation is needed to achieve the ideal capillary force. Due to the collapse of the sintered body during sintering, it was challenging to achieve bigger porosity than 60%. It is thought to need more effort to achieve the reduced effective pore size.

## 4. Conclusions

1. The necks between copper powder were formed at a sintering temperature higher than 900 °C and a hydrogen-reducing environment was also found to be necessary for the sintering of copper powder;
2. The porosity, permeability, and capillary force increased with increasing pore-former contents ranging from 15 wt.% to 40 wt.%;
3. The 100U sample with pore-former contents ranging from 30 wt.% to 40 wt.%, which has an average particle size of 100 μm and has higher porosity and permeability than the 100D and 200D samples, which should be due to the 100U sample’s better size uniformity;
4. The 200D sample, which has an average particle size of 200 μm, has lower porosity and permeability than 100U. Yet, it has the highest capillary force, which should be due to the small effective pore radius;
5. The porosity, permeability, and capillary force were significantly influenced by the powder’s particle size uniformity. When coarse and fine particles were mixed, the sintering density increased and the porosity and permeability were lowered. However, when it comes to capillary force, the mixture of variously sized particles inside reduced the effective pore radius, which resulted in an increase in the capillary force.

## Figures and Tables

**Table 1:** The amounts of pore-forming agent added to the powder mixture in terms of weight and volume.

wt.%	15	20	25	30	35	40
vol.%	39	48	55	61	67	71

**Table 2:** Average effective pore radius, porosity, and measured permeability of three different types of Cu powders.

Cu Powder Sizes	100D	100U	200D
Average effective pore radius (μm)	171.54	241.31	155.73
Porosity (40 wt.%)	53.62	59.07	56.47
Permeability ΔP (40 wt.%)	0.56	0.52	0.54
